# An Outlook of Recent Advances in Chemiresistive Sensor-Based Electronic Nose Systems for Food Quality and Environmental Monitoring

**DOI:** 10.3390/s21072271

**Published:** 2021-03-24

**Authors:** Alishba T. John, Krishnan Murugappan, David R. Nisbet, Antonio Tricoli

**Affiliations:** 1 Nanotechnology Research Laboratory, Research School of Chemistry, College of Science, The Australian National University, Canberra 2601, Australia; alishba.john@anu.edu.au; 2 Laboratory of Advanced Biomaterials, Research School of Chemistry and the John Curtin School of Medical Research, The Australian National University, Canberra 2601, Australia; david.nisbet@anu.edu.au; 3 Nanotechnology Research Laboratory, Faculty of Engineering, The University of Sydney, Camperdown 2006, Australia

**Keywords:** electronic nose, artificial olfaction, chemiresistive, selectivity, sensor array

## Abstract

An electronic nose (Enose) relies on the use of an array of partially selective chemical gas sensors for identification of various chemical compounds, including volatile organic compounds in gas mixtures. They have been proposed as a portable low-cost technology to analyse complex odours in the food industry and for environmental monitoring. Recent advances in nanofabrication, sensor and microcircuitry design, neural networks, and system integration have considerably improved the efficacy of Enose devices. Here, we highlight different types of semiconducting metal oxides as well as their sensing mechanism and integration into Enose systems, including different pattern recognition techniques employed for data analysis. We offer a critical perspective of state-of-the-art commercial and custom-made Enoses, identifying current challenges for the broader uptake and use of Enose systems in a variety of applications.

## 1. Introduction

Olfaction is one of the most complex senses possessed by humans. From the second half of the 20th century, researchers have proposed the idea of creating an artificial olfactory system, commonly known as an electronic nose (Enose), to detect and distinguish between structurally similar volatile organic compounds (VOCs). VOCs are organic compounds having high vapour pressure and low water solubility. Featuring the capability to quantify concentrations from parts per billion (ppb) to parts per trillion (ppt), Enoses have the potential to replace canines, the current gold standard for portable vapour detection [1,2].

**Figure 1:**
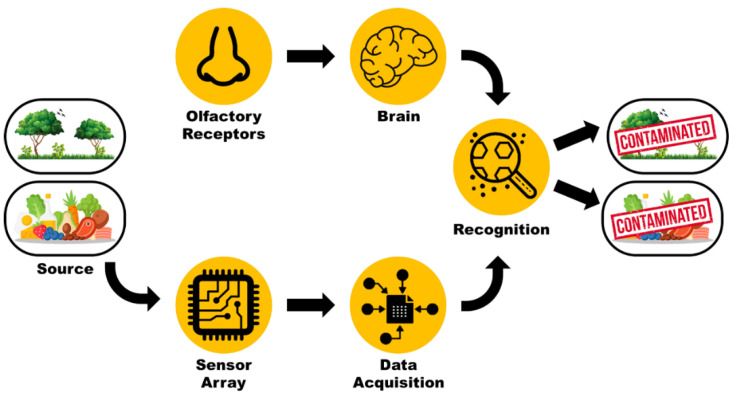
Schematic representation of the similarities between a mammalian olfactory system and an Enose.

**Figure 2:**
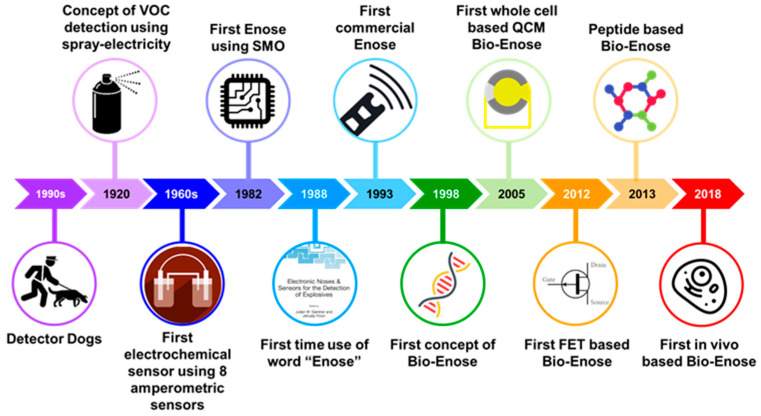
Major milestones in the development of Enose systems. VOCs: volatile organic compounds; SMO: semiconducting metal oxides; Enose: electronic nose; Bio-Enose: bioelectronic nose; QCM: quartz crystal microbalance; FET: field effect transistor.

The earliest knowledge of an artificial mechanical nose was suggested by Zwaardemaker and Hogewind in 1920, but they were unable to develop the system due to the lack of appropriate electronic and computational infrastructure [3]. In 1964, Wilken and Hartman developed the first sensor array with eight different amperometric electrochemical gas sensors composed of microelectrode–electrolyte interface, which, on application of a low direct current (DC) potential, results in a polarization reaction and produces different response patterns. However, they were unable to process these patterns [4,5]. Around the same time, Moncrieff developed a single thermistor with non-specific coating to differentiate between odours. From his observations, he postulated that utilization of six thermistors with different non-specific coatings would enable the instrument to easily differentiate between vapours [6]. This idea was further developed 20 years later by Persaud and Dodd in 1982, who developed the first Enose system using semiconducting transducers that could discriminate various odours with high sensitivity and specificity [7]. Their proposed Enose worked on a similar principle as the mammalian olfactory system. Much like humans have partially selective receptors, their Enose used an array of partially selective gas sensors that generated a certain pattern when in contact with a specific gas mixture composed of different VOCs. These patterns were characterized to identify the presence and the concentrations of analytes and their respective odours using suitable algorithms featuring similarities to the mammalian olfactory system (Figure 1). They later developed the first commercial Enose system in 1993, and since then, numerous Enoses based on semiconductors are available in the market. Despite high sensitivity towards target odorants, Enoses lack specificity. In order to overcome this challenge, a new concept of “bioelectronic nose (Bio-Enose)” was introduced for the first time by Gopel et al. in 1998 [8]. Researchers have been motivated to explore the use of olfactory receptors along with different transduction mechanisms to develop advanced Bio-Enoses [9,10,11,12]. Figure 2 describes some of the milestones achieved in the Enose technology over the last 100 years.

Conventionally, gas mixtures have been analysed using techniques such as gas chromatography (GC) [13], ion mobility spectrometry (IMS) [14], and proton-transfer-reaction mass spectrometry (PTR-MS) [15]. These approaches provide qualitative and quantitative analysis of vapours but require expert personnel and have high operational cost and time consuming sample preparation and analysis [16]. Furthermore, they are also bulky and need frequent calibration [2]. This has motivated significant efforts for the development of Enoses that may offer a more rapid, user-friendly, miniaturized, non-destructive, and affordable detection of several important gases with high selectivity and sensitivity. Some of the industrial application of Enoses include healthcare [17,18,19], food [20,21,22], environmental monitoring [23,24,25], security and military [26], and toxic gas detection [27].

**Figure 3:**
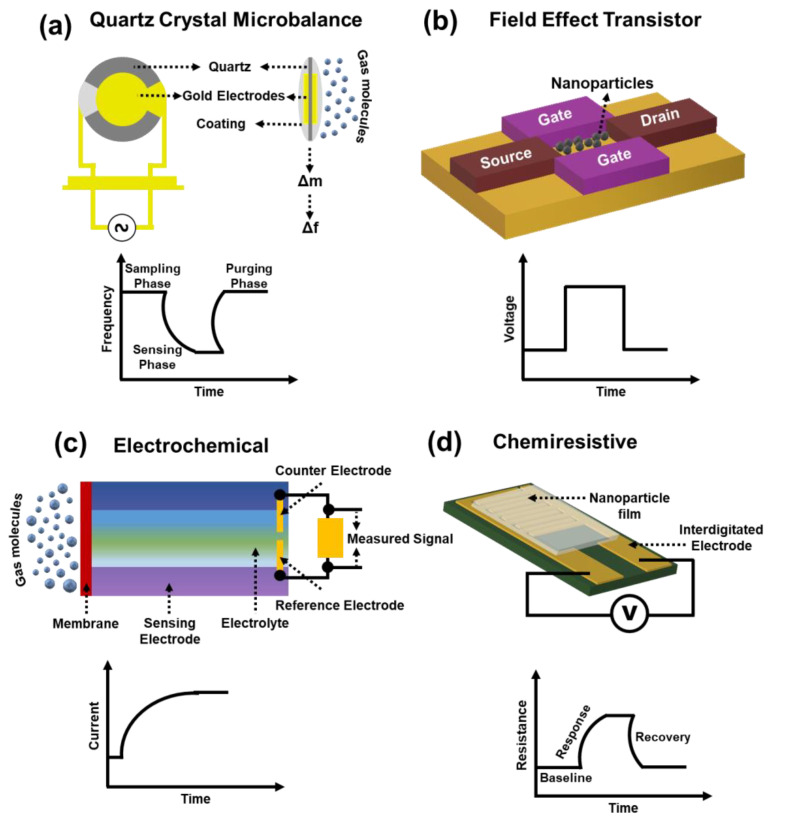
Simplified schematic illustrations of different transduction mechanisms utilized in Enoses systems: (**a**) QCM, (**b**) FET, (**c**) electrochemical, (**d**) chemiresistive.

The state-of-the-art methodologies (Figure 3) adopted for the development of Enose systems can be broadly classified into four categories: electro-acoustic sensors [28], field-effect transistor (FET) [29], electrochemical [30], and chemiresistive sensors [31]. Among the various electro-acoustic sensors, quartz crystal microbalance (QCM) is widely used in Enose systems. QCM based gas sensors rely on the Sauerbrey’s equation, where the adsorption and desorption of gas molecules on the mass of the transducer system provides a shift in resonant frequency. QCM based Enose systems offer several advantages such as real time measurements [32] and fast response [33], however, there is still room for improvement in terms of sensitivity, specificity, and portability [34].

FET-based sensors respond to the target gas by either inducing a charge transfer or modifying the nature of the conductive channel to alter the electrical characteristics (mobility, voltage) [35].

Electrochemical sensors can further be classified into four categories: amperometric, impedometric, equilibrium potentiometric sensor (EPS), and mixed potentiometric sensor (MPS). Amperometric gas sensors contain a working, a reference, and a counter electrode, which are connected via an electrolyte. When the target gas/analyte is introduced into the electrolyte either directly or with the help of a transport barrier, current changes are monitored as a function of potential to build the analytical utility [36]. EPSs can be operated either by directly measuring the mobile components or indirectly measuring the immobile species in a solid electrolyte. The sensor operates upon the deposition of the reference and the working electrode on either side of the solid electrolyte and by measuring the difference in potential between them [37]. Commonly used electrolytes for this type of sensor are yttria-stabilized zirconia (YSZ), Li_3_PO_4_, and Na_1+x_Zr_2_Si_x_P_3-x_O_12_. MPSs are based on the competing catalytic reactions between the sensing material and the target gas and between the target gas and O_2_. Responses of the sensor are measured when the rate of both the reactions are equal. Impedometric gas sensors are similar to the previously mentioned techniques, however, the sensor response between the working and the reference electrode is measured in terms of impedance instead of voltage. Response is then analysed using the equivalent Randles circuit.

Chemiresistive gas sensors function on Ohm’s Law; upon the catalytic reaction between the sensing material and the target gas, results in the change of the resistance in the setup, thereby generating a voltage signal. Amongst the different methodologies, chemiresistive is often preferable owing to advantages such as ease of fabrication, high sensitivity, and low cost and is the focus of this review.

## 2. Chemiresistive Gas Sensors

Chemiresistive sensors usually employ a two-electrode system where the resistance of the sensing layer in between the electrodes is monitored as a function of time upon gas exposure. This sensing layer can be composed of different materials such as conducting polymers [38], carbon-based materials [39], and semiconducting metal oxides [40].

### 2.1. Conducting Polymer

The use of conductive polymers (CP) and its derivatives such as poly (3,4-ethylenedioxythiophene) (PEDOT), polypyrrole (PPy), polyaniline (PANI), and polythiphene as the active layer in gas sensors dates back to the early 1980s [41] and has found significant use in several applications [38,41,42,43]. When a redox-active gas interacts with the CP, it undergoes doping/de-doping, resulting in redox reactions. These redox reactions cause a change in the resistance and the work function of the active layer, therefore facilitating the detection of different gas molecules.

Gruber et al. developed an Enose using four different polymers for the detection of *Pencillium digitatum* in post-harvest oranges [44]. The polymers used in the fabrication were poly (9,9-dioctyl-2,7-fluorenyleneethylene), poly (2,5-biphenyleneethylene), poly (4′-hexyloxy-2,5-biphenyleneethylene) and poly (2-bromo-5-hexyloxy-1,4-phenylenevinylene), each doped with 10% *w*/*w* p-dodecylbenzenesulfonic acid. The Enose developed by Gruber and co-workers was able to successfully detect early bio deterioration caused by *Pencillium digitatum* in oranges. Even though CP provides rapid response and good sensitivity, due to lack of variety and difficulty in CP fabrication, non-uniformity in physical properties, low conductivity, and lack of stability, they are not an ideal choice of sensing material for Enoses.

### 2.2. Carbon-Based

Graphene and carbon-nanotubes (CNT) have been widely used as carbon-based sensing layers for gas sensors. Owing to their structure, gas sorption is the predominant reaction taking place between the target gas and the active material [45]. The interaction between gas molecules and carbon-based materials results in charge transfer between the sensing layer and the gas molecules, thereby changing the conductivity of the material and enabling detection of various concentrations of gas [46]. Although sensitivity can be improved by functionalizing and introducing surface defects, some of the disadvantages associated with carbon-based sensing layers include lack of selectivity, long response and recovery time, and its inability to distinguish between target gases with low adsorption energies [45].

### 2.3. Semiconducting Metal Oxides

Semiconducting metal oxides (SMO) are the most commonly used active material in gas sensors mainly owing to their low cost, robustness, and easy electronic measurement properties. This type of sensor is generally constructed on inter-digitated (IDE) electrodes where the sensor response is measured in terms of resistance changes between the two-comb-like electrodes, which is a common design used for the fabrication of IDEs. The type of SMO used as the sensing layer also plays an important role in the detection of the target analyte gas. SMO are broadly classified into n-type and p-type based on their majority charge carriers.

#### 2.3.1. Types of Semiconducting Metal Oxides

##### N-Type Semiconductors

The most commonly used SMO in chemiresistive gas sensor is tin dioxide (SnO_2_) with a wide band gap of 3.6 eV. The naturally occurring and abundantly used crystal structure of SnO_2_ for gas sensing is cassiterite. Its excellent sensing properties can be attributed to its strong oxidizing power, low cost, chemical inertness, non-toxicity, and large surface area [47]. Another commonly used SMO is tungsten oxide (WO_3_) with a band gap of 2.7 eV. Owing to good sensitivity, selectivity, rich oxygen vacancy defects, surface polarization, and modulated surface activities in detecting a variety of gases, chemiresistive gas sensors based on WO_3_ nanostructures have been widely studied [48,49]. Zinc oxide (ZnO) is another popularly used SMO in chemiresistive gas sensors. ZnO has become a good candidate for chemiresistive gas sensors due to its wide band gap (3.35 eV), chemical stability, high exciton binding energy, and tuneable transport properties [50]. Regardless of its high demand for use in gas sensors, the high operating temperatures required for use of n-type SMOs restricts their usage for practical applications. Therefore, researchers have been studying the effect of metal doping, the incorporation of noble metals, and the effect of UV activation for room/low-temperature operation [51].

##### P-Type Semiconductors

Nickel oxide (NiO) having a band gap of 4.3 eV is a widely studied p-type SMO for use in chemiresistive gas sensors. Direct electronic interface, quick response and recovery, high sensitivity, and long lifetime are the properties of NiO exploited for gas sensing [52]. CuO is another p-type SMO used for gas sensors and can either exist as cupric (CuO) or cuprous (Cu_2_O) oxide with narrow band gaps of 1.2–1.5 eV and 2.12 eV, respectively. CuO can be a promising candidate for gas sensing due to its catalytic property, low operating temperature, and high sensitivity [53]. Another type of p-type SMO used in gas sensors is Co_3_O_4_ with an indirect band gap (1.6–2.2 eV) [54]. Co_3_O_4_ nanostructures′ gas sensing performance depends greatly on their assembly, crystal size, and crystal orientation [55,56]. Although p-type SMOs have lower response compared to n-type SMOs via chemical and electronic sensitization, their gas sensing ability can be significantly enhanced. Some of the methods explored to improve sensing properties include introduction of dopants, formation of p-n junctions and control of morphology.

#### 2.3.2. Sensing Mechanism

**Figure 4:**
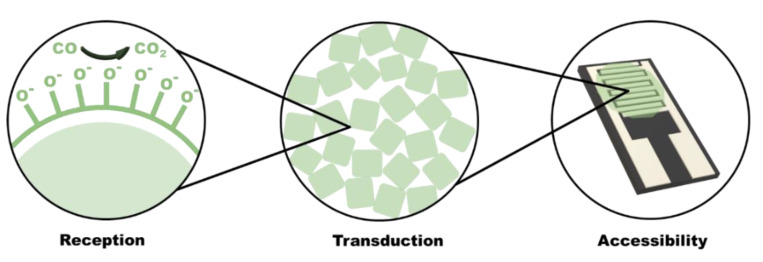
Processes involved in chemiresistive gas sensing: Left—reception mechanism: corresponds to the catalytic reaction between the adsorbed oxygen ions on the surface of the sensing material and the analyte molecules and relates with sensitivity and selectivity of the sensor. Centre—transduction mechanism: responsible for the conversion of change in reactive energy to readable device resistance. Right—accessibility mechanism: describes variation due to diffusion of analyte molecules within the grain boundaries of the SMO layers.

Irrespective of the type of SMO used as the active layer, the gas sensing mechanisms in an SMO based chemiresistive gas sensor are primarily due to the catalytic interactions on the sensing layer as well as the diffusivity of target gas molecules into the system. The sensing mechanism is dependent on reception, transduction, and accessibility to grain boundaries (Figure 4). Reception is responsible for the conversion of a chemical reaction into energy, and transduction converts the energy into a readable analytical signal.

The interaction between the target gas and the SMO layer proceeds via a reversible redox reaction, subsequently changing the electrical properties of the SMO layer. These electrical properties are translated into an appropriate readout, such as resistance measured via a transducer on an IDE.

##### Reception Mechanism

The main function of the reception function is to promote catalytic reactions between the analyte gas and the oxide surface via two reactions:

1. Ionosorption of the molecular oxygen from air and formation of reactive oxygen species (O_2_^−^, O^−^, O^2−^) on the SMO surface. These ionosorbed oxygen trap electrons from the conduction band, resulting in band bending and formation of a depletion layer along with an increase in the work function of the grains. This reaction proceeds according to the following equation [57]:
  (1) $$\frac{\beta }{2}O_{2}^{\mathrm{gas}}\;+{\;\alpha e}^{-}\;+\;S↔O_{\beta \left(S\right)}^{\alpha^{-}}$$
  - where O_2_^gas^ represents the oxygen molecules available in the atmosphere
  - e is the electron that can reach the surface of SMO; S is the unoccupied sites available for the chemisorption of oxygen molecules
  - $O_{\beta \left(S\right)}^{\alpha^{-}}$ is the chemisorbed oxygen on the surface of SMO (where α = 1 or 2 for single of doubly form; β = 1 or 2 for atomic or molecular form)
2. Reaction of the analyte gas with reactive oxygen species. This results in the formation of volatile reaction products. As the adsorbed reactive oxygen species are consumed in the reaction, it releases the trapped electrons from the SMO surface and subsequently decreases the work function of the grain [58,59].

##### Transduction Mechanism

**Figure 5:**
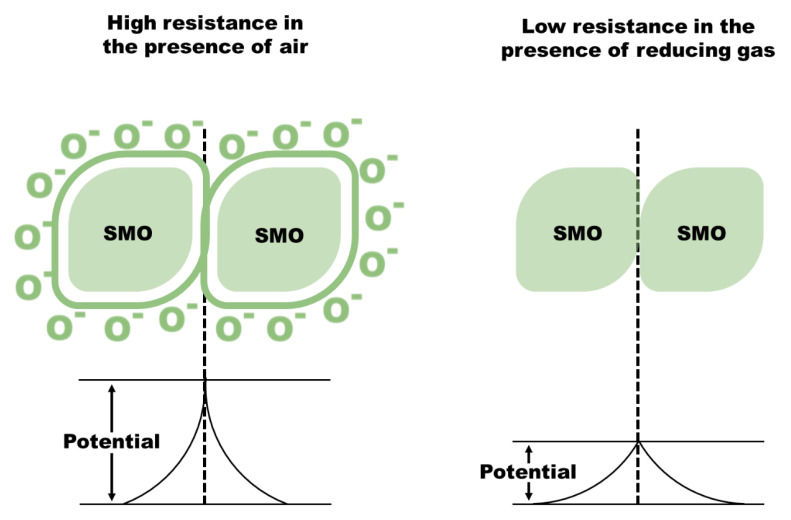
Change in charge carrier concentration in an SMO (n-type): Left—depletion layer formation (causes high potential barrier) on the surface in the presence of air. Right—reaction of target (reducing gas) with O_2_ reduces potential barrier.

In order to read the change in work function of the grains, a transducer converts it into a measurable electrical signal. The most common type of transduction mechanism adopted in SMO based chemiresistive gas sensors is measurement of DC resistance of the sensing layer. The transduction function is explained through the “double Schottky barrier model” [60]. The formation of the Schottky barrier is a resultant on the formation of the depletion layer at the surface of SMO and grain boundaries (Figure 5).

The structure and the morphology of the SMO layer play vital roles in the transduction mechanism. Due to the structural proximity of the grain boundaries, transduction can proceed either via grain boundary contact or through sintered necks [57]. Along with the microstructure, the transduction mechanism is also dependent on chemical, electrical, size, and shape of SMO [59,61] together with the electrode geometry [62].

##### Accessibility to Grain Boundaries

Accessibility of the analyte gas to the inner grains and the position of reactive oxygen species within the layer of SMO is important for gas diffusion reactions. Relative to the diffusion rate, if the reaction rate is much faster, the gas molecules are mainly adsorbed at the outer surface and do not interact with the inner sites. During the sensing process, this leaves the oxide grains on the inner sites unused, which eventually contributes to a reduction in sensitivity [63].

In the presence of multiple intervening gases, the main drawback of metal oxide gas sensors is their lack of selectivity against a single analyte gas. Researchers have constantly been seeking to find ways to enhance the selectivity of chemiresistive gas sensors based on SMO. Using sensor arrays/Enoses is one of the approaches used to enhance selectivity. Components, advantages, and certain applications of Enoses over single SMO sensor are discussed in the further sections.

## 3. Enose Systems

**Figure 6:**
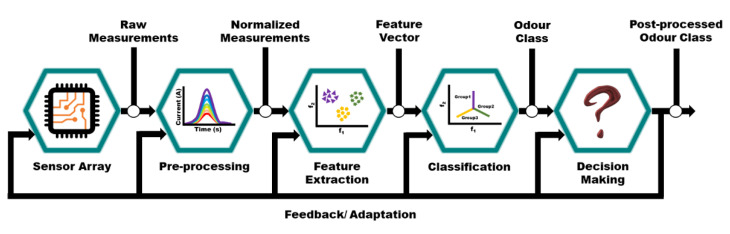
Block diagram representing signal processing architecture in an Enose. Pre-processing: collects data and converts to specific pattern. Feature extraction: extraction of robust and fingerprint information. Classification: grouping the extracted information into classes. Decision making: analysis of type and concentration of vapours.

An Enose is composed of a sample collector, a sensor array, a data processor, and a pattern recognition system. The sample collector collects the VOCs from the source to the sensor array, which then converts the chemical signal to corresponding electric signals. The selectivity of an SMO based gas sensor can be improved with the help of a data processor and various pattern recognition techniques. As shown in Figure 6, data analysis by an Enose can be represented in four stages: data acquisition, feature extraction, classification, and decision making.

Data acquisition/pre-processing collects and transforms data into a specific pattern vector. It also helps compensate the inherent sensor drift along with compressing transient response and reducing variations among samples. Then, the pattern vector is transferred to the next level, referred to as feature extraction. Feature extraction uses multiple transformation techniques from the input data to produce new significant feature vectors, thereby reducing dimensionality and extracting relevant information necessary for pattern recognition.

### 3.1. Pattern Recognition Techniques

A variety of graphical, multivariate, and neural network techniques are employed for pattern recognition. These techniques aim to identify, classify, and quantify different VOCs into respective classes based on specific patterns generated. Different fingerprints from known VOCs are used for generating a database and to train the system to categorize and distinguish unknown VOCs. This process can be divided into three distinct methods, as follows.

#### 3.1.1. Graphical Methods

Graphical approaches are the simplest tools for reducing the dimensionality of the data, thereby facilitating comparison between samples or VOCs from samples from those in the data bank. A sensor array which reacts to VOCs individually may produce a distinct reaction pattern for each individual form of analysis or for the mix. Each single sensor responds to a variety of gases, but a pattern of differential responses across the spectrum creates a specific pattern for each gas. The changes in the pattern generated using a different transduction mechanism corresponds to the type of VOC, while the intensity of the pattern corresponds to the concentration of VOC [64]. Some of the commonly used graphical approaches include bar plots, polar plots, hierarchical cluster analysis (HCA), and Sammon mapping. These techniques offer ease in pre-processing data, but sometimes the results can be non-conclusive due to the high dimensionality and the increased amount of data types.

#### 3.1.2. Multivariate Data Analysis Methods

Multivariate data analysis methods are based on multivariate statistics and are mostly used for datasets with more than one variable. These approaches reduce high dimensionality of different correlated variables to two or three dimensions. Different varieties of multivariate data analysis methods include principle component analysis (PCA), cluster analysis (CA), linear discriminate analysis (LDA), and canonical discriminate analysis (CDA). This type of approach for pattern recognition in an Enose system can be divided into unsupervised and supervised techniques. Unsupervised techniques are used when there is no database available. Such techniques are primarily used for distinguishing between known and unknown VOCs instead of identifying a specific VOC. The most commonly used unsupervised technique is PCA (also known as Karhumen–Loève transform); it is used for improved analysis of data clustering through reducing dimensionality, by eliminating the lowest ranking variables, while still retaining relevant information [65]. On the contrary, supervised techniques are useful to classify unknown VOCs based on specific characteristics from reference libraries. LDA is a popular supervised technique that helps locate projection to maximize the separation between samples for class-separation [66]. These supervised techniques provide easy to understand results along with revelations of structures within data that are not clearly evident. However, some of the disadvantages associated include insufficiency in methods to clearly distinguish between clusters and requirement of data standardization before performing these techniques.

#### 3.1.3. Neural Network Methods

Neural network approaches offer important connections and ties between several objects of various types that are not visible in isolated data banks. These techniques are the most evolved analytical tools available for pattern recognition and can be used to overcome drawbacks of the aforementioned techniques. Different methods in such an approach include artificial neural network (ANN) and radial basis function (RBF). ANN is the most commonly used and most evolved analytical tool. It is an algorithm that mostly resembles a biological neural network and has the following properties: activation function of neurons (kernels), network topology, and learning algorithm [67]. ANN is a complex yet adaptive model that changes its structure during the learning phase and can easily find or generate patterns between the inputs and the outputs in the data [68,69]. Different training methods are used for pattern recognition algorithms that search for identifying similarities and variations in recognised patterns contained in an analysis-specific reference library. The training method requires an adequate quantity of known sample data for training the system and comparison of unknown samples with known references, which is a very effective and efficient technique. The outcome of an ANN data analysis is typically represented as a percentage match between unknown and known elements of a sample from a data bank. Although neural network methods are the most advanced techniques available, they are computationally heavy and sometimes tend to overfit the results.

The approach chosen for data analysis is dependent on the type of input data available and the information obtained from the sensors. Using different combination of SMO based gas sensors and pattern recognition techniques, applications of Enoses in food industry and environmental monitoring are discussed below.

## 4. Application of Enoses in Food and Environmental Monitoring

### 4.1. Food Industry

Enoses have found numerous applications in different sectors of the food industry, such as: quality and spoilage monitoring, quantification/detection of various contaminants, evaluation of shelf-life, and identification of geographical origin. Traditionally, quality control and check in the food industry was carried out either by organoleptic assays by evaluating its texture, flavour, colour, and tenderness or using various analytical tools such as gas chromatography-mass spectrometry (GC–MS) and microbial cell counting. Although the latter are accurate and reliable techniques, they are quite expensive, require operation by an expert, and are extremely time-consuming, whereas the former techniques are unreliable due to variability between individuals [70]. Therefore, state-of-the-art Enose technology has been used in the food industry due to its simplicity, affordability, and good correlation with sensory panels. This section provides a general overview of different samples evaluated in the food industry using Enoses (Table 1).

#### 4.1.1. Bakery and Grains

Metal oxide based Enoses have recently found a great deal of use in quality monitoring and evaluation of shelf-life in the bakery and grains industry. Gobbi et al. assembled a sensor array using six commercially purchased SMOs to distinguish between six different types of fumonisin contaminants by detecting the organic vapours released from fungi in maize cultures [71]. With the help of partial least square (PLS) regression along with competitive direct enzyme-linked immunosorbent assay (CD-ELISA), they were able to quantify high and low contamination levels of fumonisin. Another similar work was carried out by Sberveglieri et al., where they used two different morphologies (thin film and nanowire) of commercial SMO to test the Enose in quality control and diagnosis of microbial contamination in a wide variety of food items [79]. Utilizing PCA in conjugation with GC-MS, they were able to successfully detect indigenous mould in coffee beans, assess spoilage in lactic acid bacteria, and identify coliforms in potable water by differentiating between microbial VOCs. Dutta et al. used an Enose composed of eight broadly tuned commercial tin oxide sensors to determine the shelf-life of nutraceutical-rich drop cookies [21]. They were able to assess the rancidity of fortified cookies prepared by black pepper and green cardamom extracts obtained from α-amylase-assisted supercritical carbon dioxide by utilising PCA and the Mahalanobis distance method. They found that the fortified cookies had an enhanced shelf-life of 120 days in comparison to the post-extracted cookies, which had a shelf-life of only for 80 and 40 days for black pepper and small cardamom cookies, respectively.

#### 4.1.2. Beverages

Tea and coffee are the most commonly consumed beverages in the globe. In order to maximize commercial profit, many producers and distributors have been counterfeiting these drinks. It is extremely difficult for human olfaction to distinguish between different blends of coffee or tea; therefore, it is very important to have a system that can easily distinguish between different varieties. Enoses have been widely used in the beverage industry for profiling various adulterants and identifying geographical origin of them along with quality monitoring. The use of an Enose to recognise the forgery of coffee brands that are made by mixing a mediocre grade and a high-quality coffee type was suggested by Brudzewski et al. [20]. Their sensing system consisted of six pairs of commercial SMO sensors divided into two identical arrays, where one set was the “measurement array”, while the other set was the “reference array”. With the concept of a differential Enose system and analysis done using PCA, support vector machines (SVM), and analysis of variance (ANOVA), they were able to improve the selectivity in measuring small concentrations (up to few parts per million (ppm)) of forgery of coffee blends. The combination of an Enose, electronic tongue (e-tongue), and sensory evaluations were used by Kovács et al. to differentiate between five varieties of Sri Lankan black tea [75]. Their Enose system consisted of 23 distinct sensors, of which 10 were metal oxide semiconductor field effect transistor (MOS-FET), and 12 were commercial chemiresistive gas sensors based on SMOs. Using pattern recognition methods such as PCA, linear discriminant analysis (LDA), and ANOVA to evaluate data, they were able to clearly differentiate between the various forms of black tea. Identification and classification of alcoholic beverages is another important sector in the food industry where Enoses have found immense applications. Santos et al. developed a handheld wireless portable Enose composed of four commercially purchased tin oxide thin film sensors, of which one of them was doped with palladium, for real time detection of defects (acetaldehyde and ethyl acetate) found in beer [78]. They were able to identify defects in beer with the aid of PCA and ANN, however, with the developed Enose system, precise quantification of the aromatic defect was not possible.

#### 4.1.3. Fruits and Vegetables

Many VOCs are released during the ripening/rotting process of fruits and vegetables. These VOCs can provide knowledge on freshness and infestation. In comparison to conventional techniques (such as GC-MS), Enoses are a facile, rapid, and portable alternative to identify contamination and determine the quality of fruits and vegetables. Guohua et al. developed an Enose sensor array with eight commercial SMO sensors that were selective towards different VOCs for the evaluation of peach freshness [81]. Using PCA and stochastic resonance (SR) data analysis techniques, they were able to predict the propagation of microorganism during storage of peaches, as the primary VOCs emitted were ethanol and ethyl alcohol. In order to detect rotten onions during post-harvest storage, Konduru et al. developed an SMO-based Enose composed of seven commercial sensors with an ability to distinguish between acetone, acetonitrile, ethyl acetate, ethanol, methlypropyl sulphide, and 2-noanone VOCs [82]. PCA and LDA analysis showed that the Enose was able to differentiate the key VOCs released during rotting of onions in storage. A commercially acquired SMO-based Enose was developed by Ghasemi-Varnamkhasti et al. to characterise the freshness of strawberries in various types of polymer packaging [83]. The effects of polymer packages on strawberry freshness have been classified and investigated using pattern recognition methods such as PCA, LDA, and SVM. With the developed Enose, they were able to distinguish between ethylene vinyl alcohol (EVOH), polypropylene (PPP), and polyvinyl chloride (PVC) packages based on the VOCs released from them.

#### 4.1.4. Meat and Fish

Quality monitoring of fish and meat is another big sector where Enose finds its application. Tian et al. reported the development of an 18 commercial sensor-based Enose to accurately differentiate between eight chicken seasonings and four beef seasonings, using different analytical techniques such as PCA, discriminant factor analysis (DFA), and cluster analysis (CA) [85]. Güney et al. fabricated an SMO-based Enose using eight commercial sensors for the discrimination between various fish species to determine its freshness [86]. Using the parameter extraction method and sub-sampling method for analysis of the data, they found that their Enose was able to easily distinguish between various fish species and determine its freshness. In order to differentiate between commonly produced VOCs from food preservatives such as ethanol, acetone, nitrogen dioxide, and ozone, Zappa fabricated a custom-made Enose consisting of three distinct metal oxide (WO_3_, SnO_2_, CuO) nanowires that were directly synthesised on micro-hotplates [84]. For data analysis, authors used the PCA algorithm and concluded that WO_3_ was the most sensitive material to nitrogen dioxide and was able to detect down to ppb level, whereas SnO_2_ was most sensitive towards ethanol and acetone, while CuO results were less than the other two materials, yet measurable.

#### 4.1.5. Milk and Dairy

Milk and milk products are a high source of calcium and good fat. Researchers have developed Enose and pattern recognition technology for differentiation between various milk products, determination of its rancidity, and evaluation of its shelf-life. Haddi et al. developed a portable Enose consisting of six commercial SMO sensors along with extraction and pattern recognition techniques such as PCA, DFA, and multivariate analysis of variance (MANOVA) to distinguish between different types of cheese [88]. They concluded, with 96% success rate, that the Enose was able to distinguish cheese made from cow′s or goat′s milk. In order to inhibit microbial production, many vendors add antimicrobial agents (e.g., detergents) in raw milk. Addition of such substances can have adverse health effects such as kidney diseases, anaemia, and dyspnoea [103]. Therefore, Tohidi et al. created an Enose system by installing eight commercially purchased SMO sensors on a Teflon-based sensor chamber [91]. Their developed sensor along with pattern recognition and analytical tools such as MANOVA, PCA, LDA, SVM, and adaptive neuro-fuzzy interference system (ANFIS) proved to be a simple and feasible technique to recognise different levels of detergent powder in raw milk. Mu et al. developed an Enose consisting of seven commercial SMO based chemiresistive gas sensors for the identification of milk from various dairy farm sources [92]. They also used the Enose for estimating the quality of milk using various machine learning methodologies such as PCA, LDA, SVM logistic regression (LR), and random forest (RF). Using their developed Enose, they were able to distinguish between the sources and also estimate the fat and protein content in milk with 95% accuracy.

#### 4.1.6. Oils

Oxidation of oil is a major cause of its quality deterioration [104]. Reasons for oxidation could range from improper processing to inappropriate storage. Various Enoses have been developed to not only detect by-products formed due to oxidation of oil but also for determining various adulterants present in them. Lerma-García et al. fabricated an Enose array composed of six commercial SMOs along with techniques such as LDA and ANN to differentiate between five types of defects (fusty, mouldy, muddy, rancid, winey) commonly found in virgin olive oils [93]. With their developed sensor array, there were able to successfully predict the defect below 0.9% error. Haddi et al. developed an Enose that had the potential to differentiate between the geographical origins of virgin olive oil based on their VOC profile [94]. Their Enose system composed of six commercial SMOs was able to successfully discriminate between twenty-seven and identify five Moroccan virgin olive oil cluster samples based on the analysis carried out by techniques such as PCA and LDA. Karami et al. constructed a portable Enose system consisting of eight SMO sensors for investigating the oxidative degree of edible oil along with identifying the presence of adulterants [97]. In combination with CA, PCA, SVM, PLS regression, and quadratic discriminant analysis (QDA) techniques, the developed Enose device was able to successfully detect the levels of oxidation in edible oil with different levels of precision and also profile the level of different adulterants in the sample of oxidised oil compared to the non-oxidized sample.

#### 4.1.7. Spices

Spices are one of the most valued commodities since ancient times. They are known to enhance flavour and scent of food. Due to high demand, production of illegitimate spices or finding contaminants in spices has greatly affected the spice trade market. In order to curb this issue, researchers have developed Enoses that can easily analyse the quality, detect contaminants, and identify the origin of species. Huang et al. employed an Enose system to identify the botanical origin and the quality analysis of honey [98]. Their Enose system was composed of three SMO chambers equipped with eighteen sensors. Using different multivariate analysis tools such as PCA, DFA, and SVM, they were able to differentiate between the origins of honey samples. While using the PLS regression technique, the developed sensor array was used to predict various quality components such as glucose, fructose, hydroxymethylfurfural, amylase activity, and acidity. Heidarbeigi et al. developed a sensory array composed of six commercially acquired SMO sensors [99]. Their developed Enose was able to discriminate between saffron and adulterated samples with 86.87% accuracy with the help pattern recognition methods such as PCA and ANN. However, the one drawback of the developed Enose was that it could only differentiate between two samples if the adulteration level was greater than 10%. Ghasemi-Varnamkhasti et al. developed an Enose composed of eight SMO sensors in combination with analytical techniques such as LDA, PLS-DA, and parallel factor analysis with linear discriminant analysis (PARAFAC-LDA) to differentiate between cumin seeds belonging to different regions based on their VOC profiles [100].

### 4.2. Environmental Monitoring

Detection and regulation of harmful chemical emissions in the atmosphere has become a major concern for many countries across the globe. Tons of organic and inorganic contaminants are released into air, soil, and water, which has the ability to pose many health threats not only to humans but also to plants and animals. Therefore, there is a need to monitor environmental pollutants to ensure the quality of human life and the sustainability of the earth. Some of the common VOC and gaseous contaminants present in the environment are tabulated in Table 2.

#### 4.2.1. Air Quality Monitoring

**Figure 7:**
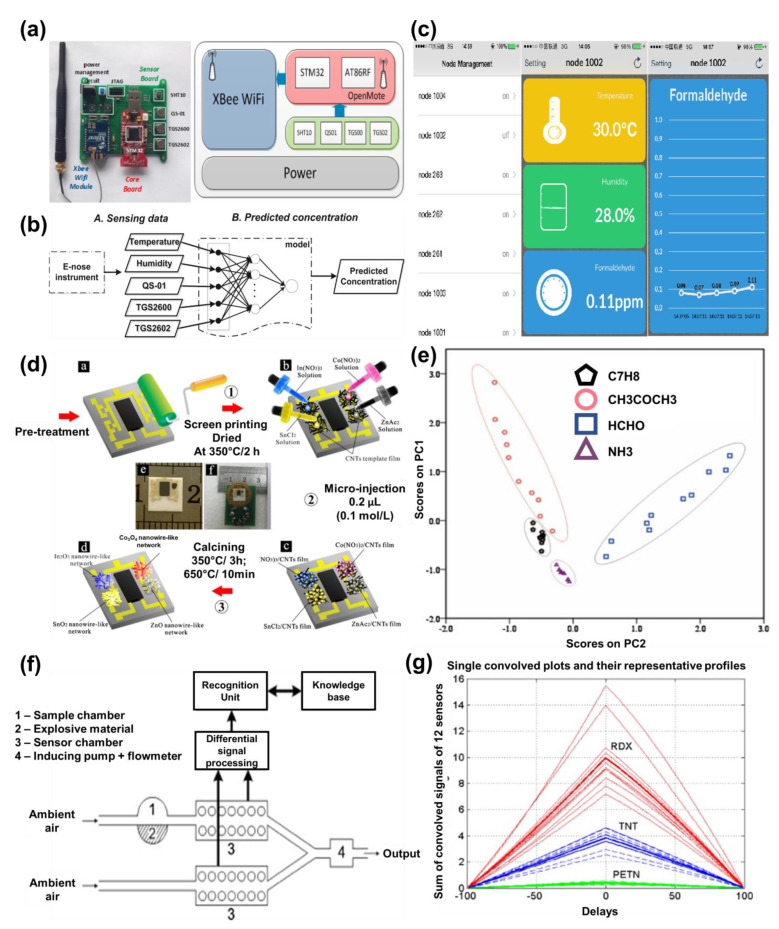
(**a**) Schematic representing the proposed Enose setup; (**b**) data processing flowchart; (**c**) mobile application interface. Reprinted with permission [105]. (**d**) Stepwise fabrication process of coplanar gas sensor array; (**e**) PCA analysis of the custom-made Enose system. Reprinted with permission [112]. (**f**) Scheme depicting differential Enose; (**g**) graphical representation of different explosives analysed and their profile vectors. Reprinted with permission [23].

In the case of environmental monitoring, sensor arrays are predominately used for air quality monitoring, analysing odour, and vapour detection from explosives. Bitter et al. prepared a multi-gas Enose sensor system consisting of thirty-eight commercially purchased tin oxide sensors integrated on a single chip for estimating and quantifying class-specific odour intensity in an indoor environment [109]. Using their multi-gas Enose system along with LDA and PLS regression analytical tools, they were able to successfully determine the air quality (in the presence of relative humidity) in an indoor laboratory environment by differentiating between vapours from seven building materials such as sealants (acrylic and silicon), wood glaze, floor adhesive, wall paint, and Oriented Strand Board (OSB). A WiFi Enose composed of eight commercially procured SMO-based gas sensors for real-time indoor air quality assessment was also created by Wongchoosuk et al. [106]. They were able to detect low concentrations of CO along with other VOCs in two separate environments (office and kitchen) with their existing Enose and PCA analysis. Leidinger et al. also developed an SMO-based Enose comprising three commercially acquired sensors to detect three target hazardous VOCs—formaldehyde, benzene, and naphthalene—to monitor indoor air quality [110]. With their developed Enose operated under dynamic temperature conditions and LDA pattern recognition technique, they were able to detect the hazardous VOCs down to ppb and sub-ppb level. He et al. assembled seven commercially purchased sensors to create a WiFi Enose system for selective detection of VOCs for indoor air quality monitoring (Figure 7a–c) [105]. Back propagation neural network (BPNN) was used as the predominant pattern recognition algorithm for analysis of the results. Their developed Enose system was able to monitor concentrations of formaldehyde (which is a popular indoor pollutant) with mean absolute percentage error (MAPE) varying between 12.18–125.34% for concentrations <1 ppm, while MAPE varied between 4.53–32.72% for concentrations >1 ppm. Prajapati et al. fabricated a custom-made sensory array consisting of four sensors using bulk micro-machining [111]. The Enose system comprising optimized thin films of ZnO, BaTiO_3_-CuO doped with 1% Ag, WO_3_, and V_2_O_5_ was used for selective detection of CO, CO_2_, NO_2_, and SO_2_, respectively, for air quality monitoring. Yi et al. also created a custom-made coplanar gas sensor array using nanowire-like network (NWN) structures of ZnO, Co_3_O_4_, In_2_O_3_, and SnO_2_ for monitoring various gases in the indoor environment [112]. They synthesized their NWN structures directly onto alumina substrates using screen printing combined with micro-injecting and calcination (SPMIC) techniques and together with a PCA algorithm (Figure 7d,e) found out that In_2_O_3_ showed the best response and had low cross-sensitivity with formaldehyde in comparison to other sensors. NO_2_ is another major air pollutant, and its exposure can have adverse short-term (inflammation in respiratory tract) and long-term (lung infections and respiratory failure) effects on humans. Sayago et al. compared the performance of a custom-made sensor array composed of three tin oxide nanofibers prepared via electrospinning against eight commercial sensors for air quality control by evaluating different concentrations of NO_2_ under various temperature conditions [113]. With the help of PLS regression analysis, they concluded that the custom-made sensor array showed better sensitivity towards NO_2_ detection with low errors compared to the commercially purchased sensor array.

Enoses can also be used for either detecting trace concentration of vapours released from explosives or for estimating the state of explosives during their storage time. Generally, the latter is more difficult to trace as the concentration of explosives are as low as ppt level. With an attempt to solve this issue, Brudzewski et al. developed an Enose with 12 commercial SMO sensors to detect and estimate the presence of TNT, PETN and RDX explosives in the storage of military objects (Figure 7f,g) [23]. With the help of PCA, their developed differential Enose was able to distinguish between the different families of explosives with 100% recognition accuracy. A custom-made Enose sensor system consisting of seven sensors was fabricated by Horsfall et al. also for the detection of vapours from explosives. Using ball milling, they synthesized various combinations of unmodified, admixed, and two-layered WO_3_ and chromium titanium oxide (CTO) to form heterojunction arrays [114]. Subsequently, synthesized metal oxide paste was screen-printed onto gold IDEs and tested against NO_2_, NH_4_, ethanol, and nitromethane for various operating temperatures. Supported by experimental results and a machine learning algorithm such as SVM, they concluded that the two-layered sensors showed higher sensitivity towards different gases such as ethanol, ammonia, and nitromethane, whereas the admixed sensors showed higher sensitivity towards nitrogen dioxide.

#### 4.2.2. Soil Quality Monitoring

**Figure 8:**
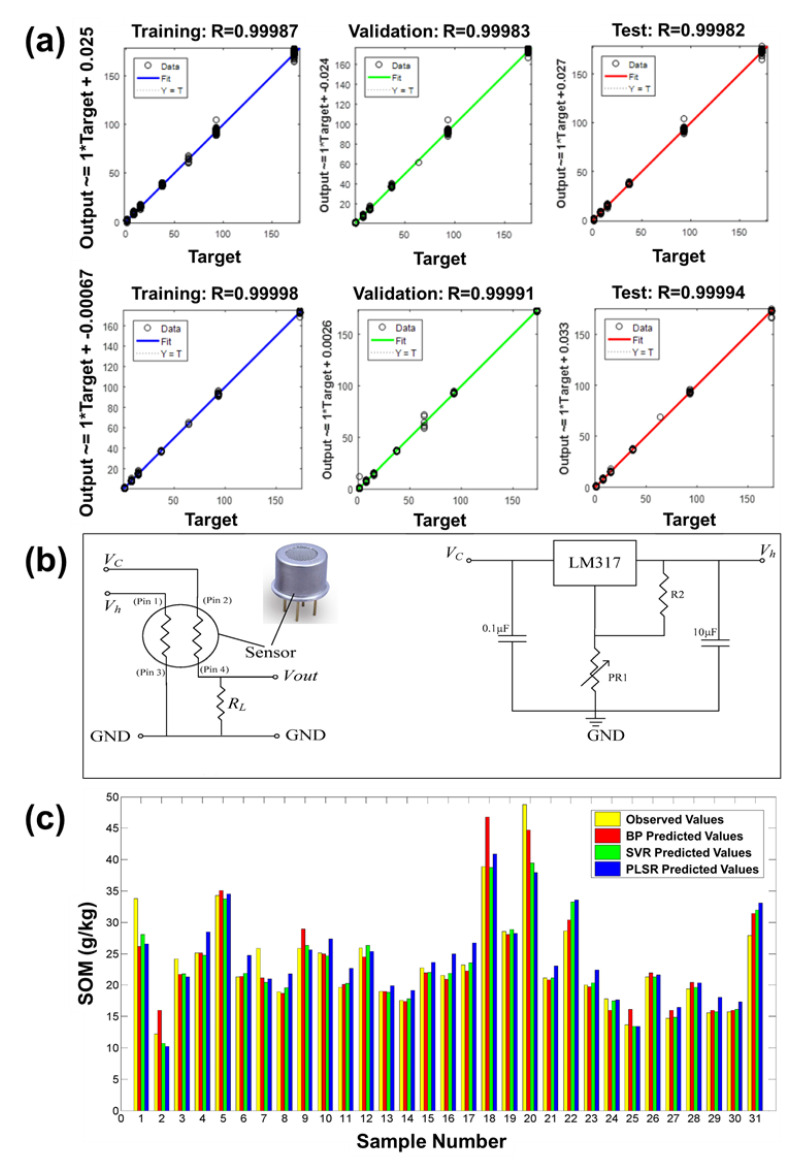
(**a**) ANN analysis for predicted and measured data sets evaluating the pollution with petrol (up) and diesel (down). Reprinted with permission [115]. (**b**) Circuit diagram; (**c**) comparison of analysed data from different models. Reprinted with permission [118].

Apart from air quality monitoring, Enoses have also been developed to detect various pollutants in soil. Rincón et al. fabricated a custom-made Enose composed of sixteen SMOs (with different combinations of SnO_2_ and TiO_2_) on a circular alumina substrate using radio frequency magnetron sputtering for in-situ monitoring of VOCs in soil contamination [25]. Using BPNN as their pattern recognition technique, they concluded that their developed Enose responded highly to oxygenated and aliphatic compounds such as propanal, methyl ethyl ketone, and octane. Eight commercially purchased SMO sensors were assembled by Bieganowski et al. to detect various VOCs for soil moisture monitoring [108]. Using analytical techniques such as PCA and ANN, they concluded that their sensor system could discern between various soil moisture conditions. The same Enose device and pattern recognition techniques were also used by Bieganowski et al. to identify levels of soil contamination caused by hydrocarbon pollution (Figure 8a) [115]. This demonstrates that a single sensor array device can be used for various applications, considering that the detected VOC profile is the same. Dorji et al. developed a portable Enose comprising eight commercially purchased sensors for soil status monitoring applicable in a precision farming system [116]. Using PCA as their analytical tool, their Enose was able to successfully fingerprint the various VOCs present in soil and also assess the grade of soil fertility. Organic content in soil plays a vital role in organic farming and is also a major indicator of soil fertility and nutrients. In an attempt to monitor the organic component present in soil, Dorji et al. also developed an Enose consisting of eight different combinations of commercially purchased SMO sensors [117]. This time, their Enose was able to successfully predict the surface soil organic matter content using pattern recognition analytical methods such as PCA and LDA, and they also concluded that the variation in VOCs emission can be attributed to the differences in light, soil temperature, and soil moisture in various areas examined. Zhu et al. also developed a sensor array system composed of ten commercial SMOs to evaluate organic matter in soil (Figure 8b,c) [118]. Their Enose system controlled at different temperatures was used to extract VOCs from soil. From the response of these sensors, four features (such as maximum value, mean differential coefficient value, response area value, and transient value at the 20th second) were extracted, and various performance parameters (such as coefficient of determination (R2), root-mean-square error (RMSE), and ratio of performance to deviation (RPD)) were analysed using pattern recognition tools such as BPNN, support vector regression (SVR), and PLS regression. They concluded that SVR has a higher predictive capability as compared to other methods and is a novel technique for predicting organic soil content.

#### 4.2.3. Water Pollution Control

Along with air and soil, researchers have also developed Enose devices for detecting various effluents in wastewater. Sensor arrays have been used to determine various parameters, such as biological oxygen demand (BOD), chemical oxygen demand (COD), total organic carbon (TOC), oxygen uptake rate (OUR), total suspended solids (TSS), pH and levels of phosphorous and nitrogen, that determine the quality of wastewater [119]. Fang et al. developed a sensor array consisting of four industrial SMO sensors for the measurement of ammonia nitrogen in wastewater using the BPNN analytical technique [120]. They were able to compute the concentration of ammonia nitrogen within a short period of time using their Enose system. Palasuek et al. developed an Enose network comprising eight commercially purchased SMO based gas sensor for monitoring the quality of wastewater [121]. Their sensor array was able to qualitatively estimate the quality of wastewater with the help of PCA, along with quantification of the BOD level. 

**Figure 9:**
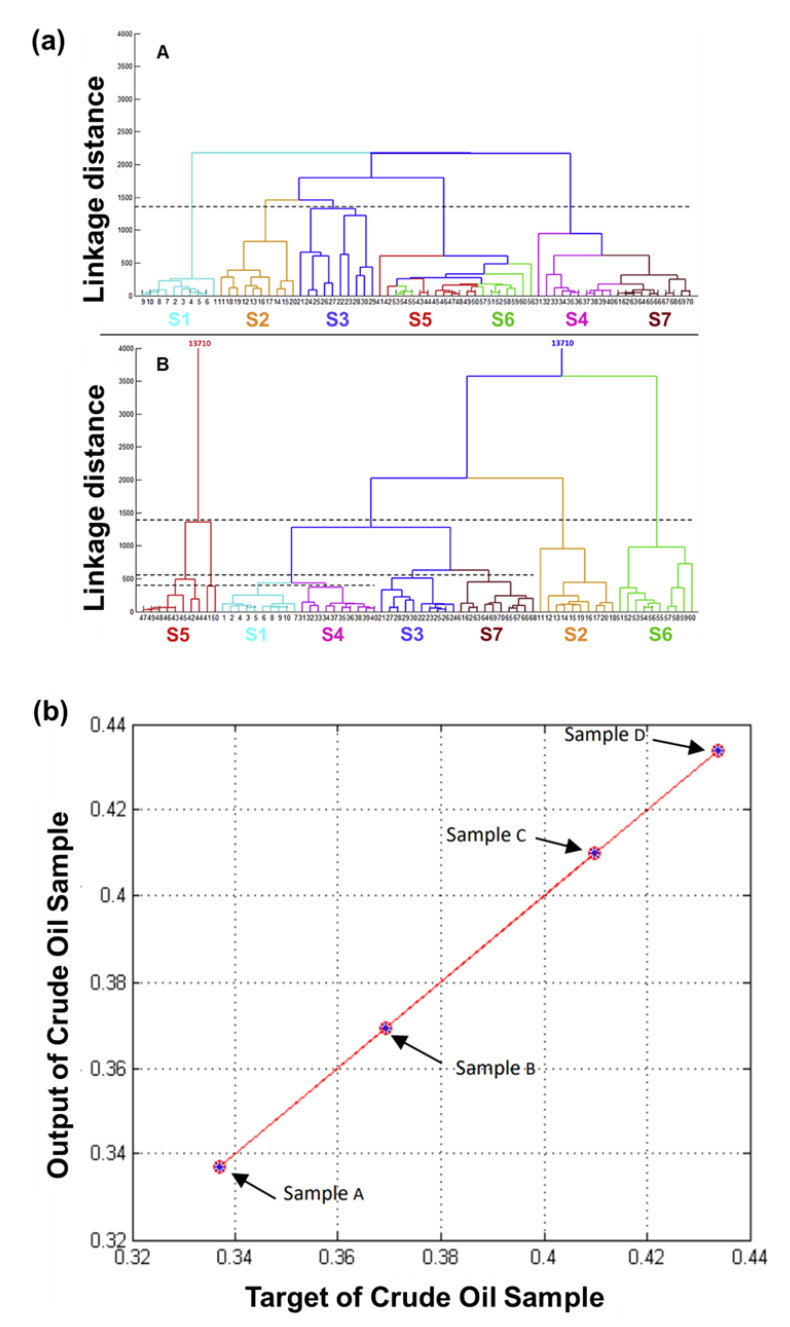
(**a**) Hierarchical cluster analysis (HCA) dendrogram obtained from air (A) and headspace (B) of the studied samples. Reprinted with permission [122]. (**b**) Regression plot obtained from waxy crude oil analysis. Reprinted with permission [24].

Moufid et al. developed an Enose platform consisting of six commercial SMO-based gas sensors in combination with voltametric e-tongue and used pattern recognition techniques such as PCA, SVM, and HCA to investigate its ability to distinguish between contaminated and clean samples (Figure 9a) [122]. Samples were also tested using traditional thermal desorption gas chromatography-mass spectrometry (TD-GC-MS) and solid phase micro extraction gas chromatography-mass spectrometry (SPME-GC-MS) to better assess the findings obtained from Enose and e-tongue. They concluded that the results obtained from the e-tongue were more accurate and precise for wastewater analysis. Nicolas et al. assembled an Enose device consisting of six commercial SMO sensors in a Polytetrafluoroethylene (PTFE) chamber with an attempt to predict the impact of odour near a waste treatment facility [123]. With the help of DFA and PLS regression techniques, they were able to sufficiently identify the five components of odour annoyance: frequency, intensity, duration, offensiveness, and impact of the receptor. However, some of the drawbacks associated with their developed Enose system included uncertainty in the calculation of the odour due to its dependence on various weather conditions and inconvenient compilation process of the results.

Contamination of building materials by microbes is another environmental issue addressed by researchers. Suchorab et al. developed an Enose using eight commercial SMO based gas sensors to detect various microbial VOCs released by ten different genres of fungi [124]. Using PCA in conjugation with chromatographic analysis, they concluded that their sensor array was able to differentiate between contaminated and non-contaminated samples and also identify individual fungi genera after 3 h of inoculation, whereas chromatography was able to differentiate between different genres of fungi only after 72–120 h of fungal growth. Mahmodi et al. developed an Enose to detect various disel-biodisel blends using eight commercial sensors and various pattern recognition techniques such as LDA, SVM, and QDA [107]. They concluded that their sensor array demonstrated superior efficacy and precision in distinguishing and classifying between different sources of biodiesel output at 87.1–94.8% accuracy using different analytical methods. 

Deposition of waxy crude oil on pipelines and reservoirs during transportation is a major issue and it also hinders the productivity of crude oil. As visual analysis of different types of crude oil deposition is cumbersome, Mawardzi et al. developed an Enose device made up of four commercial sensors to classify between four types of crude oil from different fields at room temperature based on their odour profile by differentiating between CO, LPG, CH_4_, natural gas, propane, methane, i-butane, hydrogen, and smoke (Figure 9b) [24]. Using box plot analysis and k-nearest neighbour (k-NN) techniques, they were able to differentiate between the different types of crude oil odour with 100% accuracy and 0% error.

## 5. Challenges and Future Scope

Enose technology utilizing SMO based gas sensors is a widely studied research area. However, some standing key challenges are its inability to distinguish the target gas in the presence of various interfering analytes as well as precisely quantifying its concentration. Owing to the complexity in the chemical mixtures of VOCs, they are not easily separated or identified distinctly by an Enose but are categorized into different classes using various pattern recognition systems and compared with a reference technique. The system can be modified to identify samples of unknown quality based on an existing database and correlation with a standard method. Therefore, in order to further improve the ability of sensitive and selective detection of VOCs by an Enose, the existing sensors and pattern recognition algorithms need further development.

Another challenge is the integration of nanomaterials into the Enose system. Nanomaterials have been functionalized with different organic moieties to generate specific responses to different VOCs. However, these organic moieties have a tendency to degrade over time on application of temperature or bias. Direct deposition or utilization of physical interaction between the electrode and the organic molecules can help preserve the lifetime of the sensor. Additionally, it is important to consider the following before deciding upon the best sensing nanomaterial: high sensitivity and selectivity towards target analyte, low operating temperature, rapid response and recovery times, low fabrication cost, less dependence on environmental (humidity) conditions, and stability. Incorporation of hetero-structures or new morphologies can enable new pathways for improving these challenges.

Lack of stability due to long-term and short-term drifts is another commonly faced drawback of SMO-based Enose systems, leading to difficulty in comparing data obtained at different periods. One way to tackle this impending problem is the usage of highly advanced computational algorithms. Another issue with SMO Enoses is the flooding of response where exposure to high concentrations of analyte gas molecules can sometimes lead to sensor poisoning, resulting in slow recovery. This may be improved with the usage of nanomaterials as sensing agents [125].

Another challenge that Enoses bring with the usage of many individual sensor elements is the issue of malfunctioning of one element in the whole sensor array system. This causes the Enose to fail, as most data analyses do not take this into consideration for their predictive algorithms. As a result, this requires implementation of a new Enose or a fresh algorithm, which are both expensive and time consuming. A fail-safe system built into Enoses will make them more robust. More research is required in implementing robust computational systems in order to increase the usage of Enoses for commercial applications.

## 6. Conclusions

The combination of gas sensor arrays along with data acquisition systems provides Enoses with a potential for numerous applications in the food industry to differentiate between adulterants and establish quality. It can also be employed for environmental quality monitoring and detection of explosives and hazardous gases, paving a path for enhancement in the quality of life and ensuring public safety. Among different types of sensing materials and transduction mechanisms, chemiresistive SMO nanomaterials have shown potential for multiplexing, label-free, and real-time detection. Researchers have developed Enose systems either using commercially available or custom-made gas sensors for detection of various kinds of vapours. The majority of Enose systems consist of 3–38 sensors in the sensor array. In general, a minimum of 5–6 sensors are typically needed within an Enose to differentiate between the various groups of VOCs. Greater numbers of sensors increase sensitivity and selectivity but also increase cost and decrease portability. A great deal of research has gone into an assembly process where commercially available sensors are procured and then put together to form a custom-made device, thereby only needing to work on the analysis techniques for the development of Enoses. This is mainly due to the limitations in nanofabrication of multiple sensors on a small footprint together with the lack of facile deposition techniques to grow sensing layers on each individual sensor, which inherently limits the development of Enoses. The authors direct more research into this area to further fuel the development of advanced Enoses for improved quality of life. Choosing an appropriate data processing technique is also vital for proper analysis of data. Recently, there is an increased use of neural network analytical tools and neuro-fuzzy methods, as they have the capability to reliably quantify imprecise sets of data by eliminating drift and noise from sensors. Therefore, with the advancement in electronic architecture and computational infrastructure, the prospect of integrating these sensor systems into smaller devices with higher performances will be the driving force behind the research for commercial applications.

## Figures and Tables

**Table 1:** Overview of various Enoses used in food industry *.

Category	Purpose of Analysis	Manufacturer	No. of Sensors	Material	Pattern Analysis Technique	Ref.
Bakery and Grains	Shelf life	Figaro, Inc	8	SnO_2_ based	PCA	[21]
	Contamination	SACMI IMOLA scarl, Imola, Italy	6	SnO_2_; WO_3_; SnO_2_-Au; SnO_2_-Ag; SnO_2_-Mo; SnO_2_-SiO_2_	PCA	[71]
		Alpha Soft Fox 2.0, Alpha M.O.S, France	18		PCA; MLR	[72]
	Quality	SACMI IMOLA scarl, Imola, Italy	6	SnO_2_; WO_3_; SnO_2_-Au; SnO_2_-Ag; SnO_2_-Mo; SnO_2_-SiO_2_	PCA	[73]
	Quality	Figaro, Inc	8	SnO_2_ based	LDA; SVM; KNN; RF	[74]
Beverages	Contamination	Figaro, Inc	12	SnO_2_ based	PCA	[23]
	Geographical Origin	Applied Sensor A.G., Sweden	10 MOSFET;12 MOS	–	PCA, PLS	[75]
	Identification	Figaro, Inc	8	SnO_2_ based	PCA; LDA	[76]
	Quality	Airsense Analytics, GmBH, Schwerin, Germany	10	–	ANOVA; PCA; CA	[77]
		SILSENSE	4	SnO_2_; Pd-SnO_2_	PCA; ANN	[78]
	Contamination	Figaro, Inc	5	SnO_2_-MoO_3_; SnO_2_-MO; SnO_2_	PCA; LDA	[79]
Fruits and Vegetables	Identification	Airsense Analytics, GmBH, Schwerin, Germany	10	–	–	[80]
	Quality		8	SnO_2_	PCA; SR	[81]
		Figaro, Inc. and FIS Inc.	7	SnO_2_, WO_3_	PCA; LDA	[82]
		Figaro Engineering, Inc (USA).; Hanwei Electronics Co. (China); FIS (Japan)	8	SnO_2_ based	PCA; LDA; SVM	[83]
Meat and Fish	Quality	Home-made	3	ZnO; Mn doped ZnO; F doped ZnO	PCA; SVM; DBN; Auto-encoder	[22]
	Contamination	Home-made	3	WO_3_; SnO_2_; CuO	PCA	[84]
	Identification	Alpha MOS Toulouse, France	18	Cr_2-x_Ti_x_O_3-7_; WO_3_; SnO_2_	PCA; DFA; CA	[85]
		Figaro Inc. (USA)	8	SnO_2_ based	parameter extraction; sub-sampling	[86]
		Arduino Mega	8	–	–	[87]
Milk and Dairy	Identification	Figaro Inc.	6	SnO_2_ based	PCA; DFA; MANOVA	[88]
		Fox 4000, Alpha M.O.S.	18	–	PCA	[89]
		Airsense Analytics, Schwerin, Germany	10	–	LDA; FDA; MLP	[90]
		Figaro Engineering Inc; Hanwei Electronics Co.; FIS Inc.	8	SnO_2_ based	MANOVA; PCA; LDA; SVM; ANFIS	[91]
		Figaro Inc.	7	SnO_2_ based	PCA; LDA; SVM; RF	[92]
Oils	Contamination	SACMI IMOLA scarl, Imola, Italy	6	SnO_2_; WO_3_; SnO_2_-Au; SnO_2_-Ag; SnO_2_-Mo; SnO_2_-SiO_2_	LDA; ANN	[93]
	Geographical Origin	Figaro Inc.	6	SnO_2_ based	PCA; LDA	[94]
	Quality	Airsense Analytics, Schwerin, Germany	10	–	CA; PCA; LDA	[95]
		Fiagaro Engineering (Japan); Ams (USA)	8	SnO_2_ based	PCA	[96]
		–	8	–	CA; PCA; PLS; QDA; SVM	[97]
Spices	Geographical Origin	Alpha M.O.S, France	18	SnO_2_; WO_3_; Cr_2-x_TiO_3+y_	PCA; DFA	[98]
	Contamination	Hanwei Electronics Co., Ltd., Henan, China	6	SnO_2_ based	PCA; LDA; PCR; PLS; ANN	[99]
	Identification	–	8	SnO_2_ based	LDA; PLS; PARAFAC-LDA	[100]
		Alpfha M.O.S.	6	–	ANOVA; PCA	[101]
	Quality	Karlsruhe Micro Nose	38	SnO_2_ based	PCA; LDA	[102]

* PCA: principle component analysis; MLR: multiple linear regression; LDA: linear discriminate analysis; CA: cluster analysis; PLS: partial least square; QDA: quadratic discriminate analysis; SVM: support vector machines; KNN: k-nearest neighbors; DBN: deep belief network; DFA: discriminant factor analysis; ANN: artificial neural network; PARAFAC: parallel factor analysis with linear discriminant analysis; SR: stochastic resonance; MANOVA; multivariate analysis of variance; RF: random forest.

**Table 2:** VOCs and gaseous contaminants present in the environment.

Sensor Element	Gas Detected	Detection Range (ppm)	Ref
MS1100	Formaldehyde, Toluene, Organic Solvent	1–1000	[105]
QS-01	Hydrogen, Carbon Monoxide, Ethanol, Ammonia	1–1000	
TGS2611	Hydrogen, Ethanol, Methane	500–1000	
TGS2600	Hydrogen, Carbon Monoxide, Methane, Ethanol, Isobutane	1–30	[105,106]
TGS2612	Isobutane, Ethanol, Methane, Propane	200–1000	[106]
TGS825	Hydrogen Disulfide	5–100	
TGS826	Isobutane, Hydrogen, Ammonia, Ethanol	30–120	
TGS2602	Hydrogen, Ammonia, Toluene, Ethanol, Hydrogen Disulfide	1–30	[105,106,107]
MQ135	Ammonia, Benzene and Sulfide Steams	10–10,000	[107]
MQ136	Sulfur Dioxide	1–200	
MQ3	Alcohol	10–300	
MQ9	Carbon Monoxide and Combustion Gases	10–1000 (Carbon Monoxide); 100–10,000 (Combustion Gases)	
TGS2620	Alcohol and Organic Solvents Steam, Isobutane, Hydrogen, Ethanol, Methane, Propane	50–5000	[106,107]
TGS813	Methane, Propane and Butane, Hydrogen, Ethanol, Carbon Monoxide	500–10,000	
TGS822	Organic Solvents Steam, Isobutane, Ethanol, Methane, Carbon Monoxide, N-Hexane, Benzene, Acetone	50–5000	
TGS2600-B00	General Air Contaminants, Hydrogen, Ethanol	1–30 (Hydrogen)	[108]
TGS2602-B00	Air Contaminants, Toluene, VOCs, Ammonia, Hydrogen Disulfide	1–30 (Ethanol)	
TGS2610-C00	Butane, Liquified Petroleum Gas (LPG)	500–10,000	
TGS2610-D00	Butane, LPG (Carbon Filter)	500–10,000	
TGS2611-C00	Methane, Natural Gas	500–10,000	
TGS2611-E00	Methane, Natural Gas (Carbon Filter)	500–10,000	
TGS2620-C00	Alcohol, Solvent Vapors, Carbon Oxide, Hydrogen	50–5000	

## Data Availability

Not applicable.
